# Relationship between tetracycline antibiotic susceptibility and genotype in oral cavity *Lactobacilli* clinical isolates

**DOI:** 10.1186/s13756-019-0483-9

**Published:** 2019-02-04

**Authors:** Yifei Zhang, Qian Zhang

**Affiliations:** Central Laboratory, Peking University School and Hospital of Stomatology & National Engineering Laboratory for Digital and Material Technology of Stomatology & Beijing Key Laboratory of Digital Stomatology, Beijing, People’s Republic of China

**Keywords:** Tetracycline resistance, Minimum inhibitory concentration, Whole-genome sequencing

## Abstract

**Background:**

Antibiotic resistance, is often conferred by the presence of antibiotic resistance genes. This study aimed to investigate the relationship between tetracycline resistance (Tet-R) and genotype in 31 *Lactobacillus* isolates from caries-active patients.

**Methods:**

The tetracycline susceptibility of *Lactobacillus* isolates was determined using the agar spot test and the genetic characteristics associated with tetracycline resistance using whole-genome sequencing (WGS).

**Results:**

The minimum inhibitory concentration (MIC) values of most isolates were equal to or lower than the breakpoint MIC values. Four strains that were phenotypically more sensitive (*L. fermentum* B09, S23 and *L. rhamonsus* B17) or more resistant (*L. plantarum* B43) than other isolates to tetracycline were subjected to conduct whole-genome sequencing in order to detect the tetracycline resistance genes. The results revealed that the most common Tet-R genes in *Lactobacillus* strains were *tet*T, *tet*W, *tet*O and *tet*L. In addition, *tet*PB, *tcr*3 and *otr*A were detected for the first time. There were distinct Tet-R gene mutations in *Lactobacillus* isolates. Overall, the mean expression values of Tet-R-mutated genes in *L. plantarum* B43 were elevated, and the relative expression levels of *tet*T and *tet*W genes in *L. rhamonsus* B17 *L. fermentum* B09 and S23 were decreased relative to reference strains.

**Conclusion:**

The results of this study indicate that *Lactobacillus* isolates from saliva of caries-active patients do not present considerable tetracycline resistance reservoirs. However, genetic compounds associated with tetracycline resistance were identified by whole-genome sequencing, providing meaningful insights into tetracycline resistance mechanisms.

## Background

Antibiotic resistance has become a major global public health problem [1]. It is often conferred by the presence of antibiotic resistance genes, which may spread rapidly through horizontal gene transfer via plasmids and other genetic elements, and can result the environmental contamination with antibiotic resistance strains [2]. At present, there is great concern that commensal bacterial populations, such as lactic acid bacteria from food and the intestines of animals can carry and transmit antibiotic-resistance genes [3–6]. The oral cavity represents a dynamic and complex microbial community, in which more than 700 microorganisms reside [7]. As a portal that connects the environment to the digestive tract, the oral cavity frequently comes into contact with other bacteria from the environment, and oral bacteria can easily reach other sites of the body and spread to other individuals. Therefore, oral bacteria have the opportunity to acquire and transfer antibiotic-resistant genes [8, 9]. A number of individual bacterial isolates with resistance to one or more antibiotics have been isolated from the oral cavity of both patients and healthy subjects [10–12]. The presence of genes encoding antibiotic resistance in the oral microbiota has also been recorded [13].

*Lactobacilli* are members of the lactic acid bacteria community, which can adapt to a wide variety of ecological niches (e.g., food, oral cavity, gastro-intestinal tract, and vagina) [14]. In the oral cavity, *Lactobacilli* level reflects the caries activity of an individual, since it is highly prevalent in subjects with caries-active lesions, while those who remain caries-free generally harbor low levels of *Lactobacilli* in their mouth [15, 16]. The dominant species in both adult and childhood caries include *Lactobacillus fermentum*, *Lactobacillus rhamnosus*, *Lactobacillus plantarum*, *Lactobacillus gasseri*, *Lactobacillus casei/paracasei*, and *Lactobacillus salivarius* [15, 17, 18]. Some *Lactobacilli* strains are marked as probiotics and are utilized in industrial and medical or health-related settings [19, 20]. The antibiotic resistance genes located on mobile elements (plasmids, transposons and integrons) can be transferred horizontally. As members of oral microbial communities, the *Lactobacillus* strains have the opportunity to exchange resistance factors with other microorganisms [21], potentially transferring these genes to other pathogenic bacteria. Furthermore, the emergence of *Lactobacilli* as reservoirs of antibiotic resistance genes could represent a threat to human health if antibiotic resistant oral-derived *Lactobacilli* are used as probiotic or swallowed into intestine, and this issue may exacerbated the concomitant antibiotic therapy and transfer of resistance genes to intestinal bacteria [22]. Additionally, oral-derived *Lactobacilli* have the opportunity to transfer from person to person, which could further promote the spread of a resistant strain to new hosts and subsequent dissemination of the transferable antibiotic resistance to susceptible bacteria. Thus, it is essential to check for signs of transferable antibiotic resistance in *Lactobacilli* strains that are important in oral cavity, and in strains that are used as probiotics.

Tetracycline is a broad-spectrum antibiotic that is used for the treatment of a variety of Gram-positive and -negative bacterial infections. Tetracycline resistance (Tet-R) in most bacteria is due to the acquisition of genes encoding energy-dependent efflux pumps, ribosomal protection proteins (RPPs), or enzymatic inactivation [23], many of which are related to the Tn916–Tn1545 family of transposable elements and plasmids [9, 24, 25]. The mobile elements encoding Tet-R genes are widely distributed in oral species [26–29]. Here, we conducted phenotypic and genetic analyses of Tet-R in *Lactobacilli* clinical isolates from caries-active patients.

In this study, we explored the tetracycline susceptibility of isolated *Lactobacillus* clinical strains and identified the genetic characteristics associated with tetracycline resistance using whole-genome sequencing.

## Methods

### *Lactobacillus* strains and cultivation

A total of 31 *Lactobacillus* clinical strains isolated from caries-active patients [14] were selected, containing *Lactobacillus fermentum* (*n* = 17), *Lactobacillus rhamonsus* (*n* = 7), and *Lactobacillus plantarum* (*n* = 7). Three *Lactobacillus* reference strains (*L. fermentum* ATCC 14931, *L. rhamnosus* ATCC 7469, and *L. plantarum* ATCC 8014) were used as controls. Isolates were grown in De Man, Rogosa, and Sharpe (MRS) medium (Hopebio, China) at 37 °C in an atmosphere of 5% CO_2_ and 95% air.

### Antibiotic susceptibility testing and MIC determination

An agar dilution method was used for testing the antibiotic susceptibility of isolates according to the Clinical & Laboratory Standards Institute (CLSI) guidelines, in which Mueller-Hinton medium was replaced by MRS agar [30]. For the production of the test plates, tetracycline hydrochloride stock solutions were prepared and diluted in sterile distilled water to obtain a series of tetracycline solutions with twofold concentrations from 160 to 5120 μg/mL. First, 2 mL antibiotic solution was mixed with 18 mL MRS agar to obtain test plates of the final range (2~512 μg/mL). Individual colonies of each *Lactobacillus* strain were grown in MRS broth to obtain a density corresponding to OD_630_ = 0.02 (approximately 5 × 10^8^ colony-forming units/mL). Then, 10 μL of bacterial solution was inoculated on each test plate. The plate without tetracycline was used as control. After 20~24 h incubation at 37 °C in an atmosphere of 5% CO_2_ and 95% air, the plate with lowest tetracycline concentration producing lower than thirty colony was determined and defined as the minimum inhibitory concentration (MIC) of each strain. The strains with MIC values lower than or equal to the microbiological breakpoints for anti-tetracycline (8 μg/mL for *L. fermentum* and *L. rhamonsus*; and 32 μg/mL for *L.plantarum*) [31] were defined as susceptible. Three independent biological replicates were performed.

### DNA extraction and Illumina HiSeq sequencing

According to MIC values, four *Lactobacillus* clinical isolates, which were phenotypically more sensitive (*L. fermentum* B09, S23 and *L. rhamonsus* B17) or more resistant (*L. plantarum* B43) than other isolates to tetracycline were subjected to conduct WGS to detect the Tet-R genes. First, Bacterial genomic DNA was extracted with a TIANamp Bacteria DNA Kit (Tiangen Biotech Co., Ltd., China) according to the manufacturer’s instructions. Genomic DNA was evaluated and quantified using a Nanodrop 8000 instrument and Qubit 3.0 fluorometer (Thermo Scientific, USA). High-quality DNA samples (OD_260_/OD_280_ = 1.8~2.0, > 1 μg) were utilized to construct the fragment library.

Purified genomic DNA was sheared into 300–500 bp fragments by sonication, and the library was then constructed following the Illumina TruSeq™ Nano DNA Sample Prep Kit instruction (Illumina, USA). The index tag was introduced into the adapter at the PCR stage when appropriate. The high quality Illumina pair-end library (2 × 150 bp) was sequenced with the Illumina HiSeq platform.

### Genome assembly and SNP analysis

We used ABySS (http://www.bcgsc.ca/platform/bioinfo/software/abyss) and GapCloser software (https://sourceforge.net/projects/soapdenovo2/files/GapCloser/) to perform genome assembly with multiple-kmer parameters based on the high-quality data for the final assembly results.

The MUMmer blast software was used for SNP analysis. *L. fermentum* ATCC 14931 (NZ_GG669901.1) was selected as the reference strain for *L. fermentum* clinical strain B09 and S23; *L. rhamnosus* ATCC 53013 (NC_017482.1) was selected as the reference strain for *L. rhamnosus* clinical strain B17 and *L. plantarum* ATCC 8014 (NZ_CP024413.1) was selected as the reference strain for *L. plantarum* clinical strain B43. All of the sequences were blasted with the Comprehensive Antibiotic Resistance Database (CARD).

### Real-time RT-PCR

RNA was extracted from four *Lactobacillus* clinical isolates and corresponding reference strains following the RNeasy Mini Kit (Qiagen, USA) following the instructions. RNA was quantified using Nanodrop 8000 spectrophotometer. 1 μg RNA was treated with DNase I and reverse-transcribed with ReverTra Ace qPCR RT Master Mix (Toyobo,Japan). Quantitative real time polymerase chain reaction (qPCR) was performed using PowerUp SYBR Green Master Mix in an ABI 7500 system (Thermo Scientific, USA). Primers sequences were designed and listed in Table 1. All reaction volumes were 20 μl and underwent the following reaction condition: initial denaturation for 10 min at 95 °C, followed by 40 cycles of 10 s at 95 °C and 1 min at 55 °C for fluorescence collection, with extension of 1 min at 72 °C. The bacterial 16S rRNA gene was used as a reference for calculating target gene expression. The qPCR was carried out in technical triplicates. The relative expression of *tet* genes was calculated by the 2^-ΔΔCt^ method.

**Table 1:** Primers for the detected Tet-R genes in selected clinical isolates and ATCC strains

Bacteria	Genes	Primers(5′-3′)	Amplified length (bp)
*L. fermentum*	*tet*PB	F:AACTTACCCGATGGACTGGC	146
		R:CCCCAATCACTTCCCCGTTT	
	*tet*T	F:CGACCCTATCCGAAGCCCTCT	153
		R:GGGCGTATCTAGGAGGGTGAGTT	
	*otr*A1	F:GCCGTTACCGTTAGCATTAG	195
		R:TAACTTAGCCAAAAAGGAGGGGATG	
	*otr*A2	F:ATAGCGGAGGCGTAAACTACTGGG	166
		R:TAAAGAAGGCCCTGGAACAACAC	
	*tet*W1	F:ACGCTTTGGAGTTGGGATGT	108
		R:GTTGTTCGTGGGTCCGCTCTT	
	*tet*W2	F:GAAGGTCAGGGCGGCGTCGTTT	108
		R:ATGCAGCCAATGGTCTACGC	
	*tcr*3	F:GCGTTGGCTGAAGTAAAAGATGA	177
		R:CCCCACAACGAAAACCCCCACTT	
*L. rhamonsus*	*tet*W	F:GCAAGACTGCGACTAACTTCATAAC	161
		R:GTTCTGGACGATATGGCACTTGA	
	*tet*O	F:TGGCCTTCAATTCAAGCACATCT	113
		R:AGACTGGGGTGGCGACACTATTT	
	*tcr*3	F: TAAGACGCCACTAAGCAGCAAAG	131
		R:CCATTGTCGGCGGGTATCTGTTA	
	*tet*T	F:ATCACATTCTTCGGGGTTACACG	151
		R:TACAGCGGTAGCAGGGGACATTG	
*L.plantarum*	*tet*T	F:CATTTTGAACCGTTACGACACT	260
		R:GTCGCTTCACGAAAGTCACCACC	
	*tet*O	F:CCTTTTCCACGGTCAAGACTAGC	180
		R:TCTGATTCCTGAAGATTGGGGTG	
	*tet*L	F:TAACAAGTAAGCCGTGGTCATCC	123
		R:GGATTACTTTCATTTTGCGGGGT	
	*tcr*3	F:GTCTCATTTGTTGCCGACACTTC	135
		R:TGTGCTGCCGTTTTTTGTGGTCC	
	*tet*PB	F:ACTTGGCAAACAGCGGGGACT	144
		R:CACTGACTTCATTAGCCATA	
	16 s rRNA	F:CCTACGGGAGGCAGCAGTAG	101
		R:CAACAGAGCTTTACGATCCGAAA	

## Results

### Antimicrobial susceptibility

The MIC of each *Lactobacillus* strain is summarized in Table 2. A narrow range of MIC values was exhibited by most *L. fermentum* and *L*. *rhamonsus* strains (4–8 μg/mL), except for three strains (*L. fermentum* B09, *L. fermentum* S23 and *L*. *rhamonsus* B17) with a MIC lower than 2 μg/mL and a *L*. *rhamonsus* strain B22 with the MIC value of 16 μg/mL. All *L. plantarum* strains displayed higher MIC values than *L. fermentum* and *L*. *rhamonsus,* ranging from 16 to 32 μg/mL, except for one strain (B43) with a MIC higher than 64 μg/mL. According to the microbiological breakpoints for anti-tetracycline defined in [31], only *L*. *rhamonsus* B22 and *L. plantarum* B43 displayed resistance to tetracycline.

**Table 2:** The distribution of tetracycline resistance genes and the range of MIC among *Lactobacillus* isolates and ATCC strains

Bacteria		Breakpoints for Tetracycline Resistance (μg/mL) (EFSA, 2008)	MIC (μg/mL)
*L. fermentum*	B09^a^	8	< 2
	B58		4
	B52		4
	S19		8
	B86		4
	B77		4
	S12		4
	B16		8
	B32		8
	B50		4
	B59		4
	S29		8
	S23^a^		< 2
	B82		8
	B48		4
	B84		4
	B36		4
	ATCC14931		16
*L. rhamonsus*	B17^a^	8	< 2
	B22		16
	B37		8
	B61		8
	B71		8
	S22		8
	B18		4
	ATCC53103		8
*L.plantarum*	B01	32	16
	B14		16
	B24		16
	B40		16
	B41		16
	B43^a^		64
	B68		32
	ATCC8014		16

^a^: indicates the clinical isolates for whole-genome sequencing

### Detection of putative tetracycline resistance genes

The whole genome sequences were obtained by assembling clean reads (11,590,856 reads for B09, 14,537,793 reads for S23, 19,571,983 reads for B17, and 11,723,377 reads for B43; Average coverage > 500 folds), and the results were compared with corresponding reference strains and CARD. All selected genes were identified using the following criteria of e-value <1e-20, sequence identify was higher 20% and bit scores > 50 [32]. The WGS data are available from the Sequence Read Archive under accession numbers SRR8300881, SRR8300882, SRR8300883, SRR8300884. The four isolates had the same Tet-R genes as corresponding reference strains, in terms of gene type. No gene deletion or exogenous Tet-R genes were detected. The most common Tet-R genes in *Lactobacillus* strains were *tet*T, *tet*W, *tet*O and *tet*L. Some new Tet-R genes were also detected, such as: *tet*PB, *tcr*3 and *otr*A (Table 3), which may be responsible for the molecular tetracycline resistance determinants. We also found that *tet*T, *tet*W and *tet*O genes, which have previously been investigated in *Lactobacilli* were more diverse and displayed low homology among different isolates.

**Table 3:** SNPs and relative expression of mutant Tet-R genes in selected four clinical isolates and ATCC strains

Strains	Predicted Tet-R genes	Position in Reference	SNP	Relative expression of mutant Tet-R genes
B09/S23	*tet*PB	57,351	C to T	4.18 ± 1.58*/3.87 ± 0.32*
		57,574	A to T	
	*tet*T	19,518	G to A	0.32 ± 0.21*/0.12 ± 0.04*
		20,404	T to C	
		20,503	A to G	
		21,180	A to G	
	*otr*A1	121,289	A to G	1.74 ± 0.23*/0.71 ± 0.17
		121,893	A to G	
		122,217	A to G	
		122,373	G to A	
		122,475	A to G	
	*tet*W1	201,420	A to C	7.9 ± 1.47*/1.54 ± 0.12
		201,567	G to A	
	*otr*A2	217,467	C to T	0.90 ± 0.16/1.02 ± 0.21
		217,786	G to A	
	*tet*W2	86,983	A to G	0.53 ± 0.89/0.61 ± 0.12
	*tcr*3	116,527	G to A	0.49 ± 0.03/0.65 ± 0.15
		117,173	A to G	
B17	*tet*W	1,623,933	T to A	0.43 ± 0.12*
	*tet*O	1,636,215	G to A	0.21 ± 0.02*
		1,636,258	C to T	
		1,636,464	C to G	
		1,636,637	T to G	
	*tcr*3	210,281	G to A	1.63 ± 3.10*
	*tet*T	1,850,771	T to C	0.13 ± 0.01*
		1,851,067	A to G	
B43	*tet*T	89,875	G to A	1.21 ± 0.29
		90,659	C to A	
		90,711	A to G	
		90,814	G to A	
		90,966	G to A	
		91,116	T to C	
	*tet*L	1,722,509	C to A	5.03 ± 0.95*
		1,722,558	A to C	
		1,722,936	A to C	
		1,722,984	G to A	
	*tet*O	1,810,986	C to T	2.05 ± 0.49*
		1,811,028	C to G	
		1,811,043	G to A	
		1,811,052	G to A	
	*tcr*3	1,178,583	G to A	4.99 ± 1.24*
		1,722,509	C to A	
		1,722,558	A to C	
		1,722,936	A to C	
		1,722,984	G to A	
	*tet*PB	2,686,361	T to A	2.84 ± 1.08*
		2,686,721	C to A	
		2,686,722	G to A	
		2,686,946	T to G	
		2,686,952	C to T	
		2,687,201	C to T	
		2,687,423	G to A	

* is represented the relative expression level of mutant Tet-R genes was significant difference between isolates and references strains.

To identify the changes responsible for Tet-R, we further analyzed single nucleotide variations in Tet-R genes of high-susceptible (B09/S23 and B17) and resistant isolates (B43) (Table 3). B09 exhibited 16 nonsynonymous mutations in five Tet-R genes (*tet*PB, *tet*T, *tet*W, *otr*A and *tcr*3), S23 exhibited the same variations as B09 in relation to the reference genomes (ATCC14931). Interestingly, both B09 and S23 contained a 12 bp deletion at the 122,242 position. Mapping the B17 sequence results against with the reference genome (ATCC53103) showed that there were 10 nonsynonymous mutations in four Tet-R genes (*tet*W, *tet*O, *tcr*3 and *tet*T) in strain B17; it also confirmed that B43 exhibited 22 nonsynonymous mutations in five Tet-R genes (*tet*T, *tcr*3, *tet*O, *tet*PB and *tet*L), compared to the reference genome (ATCC8014).

### Expression analysis of Tet-R mutation gene

To reveal the relationship between genetic variation and antimicrobial susceptibility, we utilized qRT-PCR to determine the expression of mutant Tet-R genes in susceptible/resistance isolates and reference strains. The relative expression levels of *tet*PB and *tet*W1 in B09 and S23; *tet*O, *tet*PB, *tet*L and *tcr*3 in B43 isolates were significantly increased compared to the respective reference strains. Also, the relative expression level of *tet*T, *tet*W2, *otr*A and *tcr*3 in B09 and S23, and of *tet*W, *tet*O and *tet*T in B17 isolate, were significantly decreased compared to the respective reference strains. The remaining detected mutant Tet-R did not differ among the above three isolates. The relative expression of most of mutant Tet-R genes in the susceptible isolates (B09/S23, B17) was down-regulated, while the relative expression in the resistance isolates (B43) was up-regulated.

## Discussion

Tetracycline resistance genes commonly found on conjugative transposons of the Tn*916*/Tn*1545* family, are easily disseminated among bacteria. Devirgiliis et al. found that the conjugative transposon Tn916 carrying the tet(M) gene can be interspecies transferred from *L. paracasei* isolates to the opportunistic pathogen *Enterococcus faecalis* [21]. Ready et al. observed the transfer of Tn916-like elements between oral *Veillonella* spp. and S*treptococci* spp. [13]. It has been reported that *Lactobacilli* present in fermented foods or animal intestines may represent an important reservoir of transferable Tet-R genes [4, 33]. However, according to other research, *Lactobacilli* clinical strains from food [34] and animal fecal microbiota [35, 36] do not present with considerable Tet-R and *tet* gene expression is rarely detected. We have reported for the first time the presence of Tet-R in *Lactobacilli* isolates derived from human saliva. With the exception of the reference strain *L. fermentum* ATCC 14931 and tested strains *L. rhamonsus* B22 and *L. plantarum* B43, all tested strains remained tetracycline susceptible. Our results indicated that *Lactobacilli* isolates from the oral cavity showed a very low prevalence of resistance to tetracycline.

We then investigated the correlation between the phenotypic susceptibility of tetracycline and the existence of Tet-R genes. The isolates investigated in this study, *L. fermentum* (B09, S23) and *L. rhamonsus* (B17) were phenotypically more sensitive (high-sensitive), while *L. plantarum* (B43) were more resistant (high-resistant) to tetracycline than other isolates. Jungermann et al. first reported the presence of *tet*W and *tet*Q in endodontic infections. They found that the *tet*M and *tet*W genes showed near-equal prevalence in root canal specimens with endodontic infections, and increased prevalence relative to *tet*Q. After treatment, *tet*W and *tet*Q were significantly reduced, with no change in *tet*M [37]. Villedieu et al. reported that *tet*W was the second most common Tet-R gene in the oral microflora of healthy adults. It has also been shown that this gene is present in oral *Lactobacillus* species [38]. Another study confirmed that the *tet*M gene was more prevalent in asymptomatic cases, while *tet*W was more prevalent in acute apical abscess cases [28]. The presence of Tet-R genes varied among different *Lactobacillus* species and in this study varied even within species in our investigation. According to previous studies, the most common Tet-R genes encoding a RPP or efflux protein, which were identified in foodborne or animal origin, were *tet*M, *tet*W, *tet*K, *tet*L, *tet*S and *tet*O [5, 39]. Also, *tet*O has been found in the plasmid of *Streptococcus mutans*, a pathogen related to caries [40]. Through whole-genome analysis, *tet*T, *tet*PA, *tet*PB, *tcr*3, *tet*W, *tet*O, *tet*L and *otr*A were detected in our isolates. As far as we know, this is the first study of *tet*PB, *tcr*3 and *otr*A that detected in *Lactobacillus* species, highlighting the values of WGS as a tool for identifying new resistance genes. We did not find any exogenous Tet-R determinant in tested strains relative to the reference strains, revealing the low possibility of the horizontal transfer of Tet-R genes in saliva-derived *Lactobacilli*.

It has been reported that mutations in *tet*A encoding tetracycline efflux pumps can reduce sensitivity to glycylcycline, a novel class of tetracycline [41, 42]. Here, we identified new mutations in *tet* genes encoding RPP (e.g. *tet*PB, *tet*W, *otr*A, *tet*T and *tet*O) that can affect gene expression. Note that in two unrelated *L. fermentum* strains B09 and S23, the exact same mutation is responsible for Tet-R, which opens possibility of developing molecular screening tests for Tet-R in *L. fermentum*. However, each strain has more than one Tet-R gene with mutation-dependent expression. Thus it is difficult to determine which Tet-R gene mutant is responsible for the altered Tet-R. In B43 isolates of *L plantarum*, the expression of all Tet-R genes were up-regulated with the higher Tet-R. Most Tet-R genes in *L. fermentum* strains B09/S23 and *L. rhamonsus* B17 had lower mean expression, consistent with their tetracycline susceptibility, thus supporting our hypothesis. The expression levels of several Tet-R genes in our study were not consistent with the genotype, which is consistent with previous reports [43]. It is likely that the resistance level conferred by different Tet-R genes is species and strain-dependent [44]. The two types of *tet* genes: active efflux and ribosomal protection, may play different physiological functions in the same isolates. Additionally, the expression of different Tet-R genes may be induced at different tetracycline concentrations [44]. Moreover, genetic mutation may influence relevant enzymatic or promoter activity [4]. However, further studies are needed to investigate other mechanisms independent of Tet-R genes that contribute to Tet-R.

## Conclusion

Taken together, our findings indicate the presence of multiple Tet-R genes in the genome of *Lactobacillus* isolates from human saliva, though most isolates do not show desired Tet-R. Several mutations in *tet* genes encoding ribosomal protection protein can affect gene expression. Exploring the mechanisms underlying this inconsistency between phenotypic resistance and genotype will be an interesting avenue for future research.

## Abbreviations

CARD: Comprehensive antibiotic resistance database
MIC: Minimum inhibitory concentration
PCR: Polymerase chain reaction
qRT-PCR: Quantitative real time- polymerase chain reaction
RPPs: Ribosomal protection proteins
SNP: Single nucleotide polymorphisms
Tet-R: Tetracycline resistance
WGS: Whole-genome sequencing
